# True sustainability is sustanimalism

**DOI:** 10.1093/biosci/biae100

**Published:** 2024-10-24

**Authors:** Pim Martens, Maarten Reesink, Karen Soeters

**Affiliations:** Maastricht University, System Earth Science, and University College Venlo, Maastricht, Limburg, Netherlands; Media Studies at the University of Amsterdam, Amsterdam, Noord-Holland, the Netherlands; House of Animals, Amsterdam, the Netherlands

The most common definition of *sustainability* is found in the Brundtland Report of 1987. *Sustainability* is described in this report as “development that meets the needs of the present without compromising the ability of future generations to meet their own needs.” It is a description that can be interpreted in various ways. So far, this has not been very favorable for animal welfare and a sustainable relationship between humans and animals. In the vast majority of policy reports on sustainable development, animals are mainly considered successful economic products and negatively as producers of methane and nitrogen or spreaders of diseases. According to various researchers, sustainability is very much determined by the relationship between humans and animals (Schlottmann and Sebo 2018), but governments and industries view sustainable development primarily through the lens of economic growth. For example, they pay little attention to animal welfare in intensive livestock farming and its consequences for public health and animal welfare. This is evident from recent outbreaks of viruses such as Q fever, avian flu, swine flu, and, most recently, coronavirus. Another worrying trend is that farm animals are given antibiotics, which enter humans through food. This is already causing antibiotic resistance in a large number of human pathogens.

These are just a few examples that show that in sustainability, our well-being, and health are closely linked to those of animals. Meat consumption, the trade in wild animals, for example, is described as “unsustainable” in sustainability reports, but this is because meat's production contributes to global warming, which, along with animal diseases, can have negative economic consequences. Such practices are not rejected because of chronic animal suffering.

The concept of sustainability dates back to the 1980s. Although a significant improvement over the earlier view that natural resources could be used indefinitely, it is still very anthropocentric: Human interests are central. As we increasingly realize that all life on Earth is interconnected and that humans certainly do not have a unique position in this, sustainability is an outdated concept.

If we want to keep the Earth livable, we must take into account the needs, feelings, and intrinsic value of all living beings, including other animals: sustanimalism (Martens 2020). Sustanimalism goes much further than sustainability in that it prioritizes animal interests and respect for all animals (not just humans) and their natural environment. We are launching this term *sustanimalism* as a call to action. We want the definition of sustainability to be adjusted to include sustanimalism from now on. Something can only be truly sustainable if it is also sustanimal.

Sustanimalism calls for a new vision of our relationship with the living (and nonliving) nature around us. A vision that not only addresses the excesses and symptoms of what is wrong with our current relationship with our fellow creatures but also constantly invites us to reflect on the fundamental ideas and values with which we view them.

And it can happen quickly. It's quite possible that serving and eating meat in public places will soon become a form of socially undesirable behavior. Maybe it will be banned. It could be that the next generation, once they are in power, will make definitive and irreversible progress toward a fossil-free economy, a circular consumption chain, and an animal-friendly, diverse, and inclusive society. The generation after them may look back in amazement and bewilderment at a time when we drove diesel cars and flew in kerosene-guzzling giants, systematically condemning other animals to miserable lives and premature deaths. They'll look back on today as a time when we still cared too little about the lives of future generations of humans and animals, when *sustanimalism* was still a new word.

## Author Biography

Pim Martens (p.martens@maastrichtuniversity.nl) *is* affiliated with Maastricht University, System Earth Science, and University College Venlo, in Maastricht, Limburg, in the Netherlands. Maarten Reesink is affiliated with Media Studies at the University of Amsterdam, in Amsterdam, Noord-Holland, in the Netherlands. Karen Soeters is affiliated with House of Animals, in Amsterdam, in the Netherlands.
